# Comparison of Three Different Methods of Dressing for Partial Thickness Skin Graft Donor Site

**Published:** 2013-01

**Authors:** Seyed Esmail Hassanpour, Seyed Mehdi Moosavizadeh, Masoud Yavari, Hamid Reza Hallaj Mofrad, Alireza Fadaei

**Affiliations:** Department of Plastic and Reconstructive Surgery, Shahid Beheshti University of Medical Sciences, Tehran, Iran

**Keywords:** Skin Graft, Donor Site, Dressing

## Abstract

**BACKGROUND:**

Split- thickness skin graft is one of the most common operations in plastic surgery. It is always painful and patient discomfort from donor site often is more significant than recipient site. There is not still a standard method for treatment of the donor site. The purpose of this study was to determine the best method of dressing the donor site among three different methods with respect to the rate of healing, pain, secretion, infection and cost.

**METHODS:**

The study includes 60 patients that were randomly divided into three groups. Donor site and thickness of the graft was the same and were dressed with one of the methods including Method A: Paraffin fine mesh gauze, Method B: Nitrofurazone soaked fine mesh gauze and Method C: Dry fine mesh gauze. Each method included an intermediate layer of sterile plastic sheet witch was covered with 10 layers of dry gauze. Comparison with respect to the rate of healing, pain, secretion, infection and cost was done.

**RESULTS:**

Thirty seven patients were men and 23 were women. The mean age of the patients was 27.2 years. There was a significant difference between three methods in average time of repair and superiority of dressing with Method B was noted. Pain severity was the least in Method B and difference between the methods was significant. Dressing with Method B had the least secretion and there was a statistically significant difference between three methods. There was no statistically significant difference in cost of the management.

**CONCLUSION:**

This study showed that dressing the donor site with nitrofurazone ointment soaked gauze used as the first layer of dressing and intermediate layer of sterile plastic sheet which was covered with 10 layers of dry gauze was the best method of dressing and had the least complications.

## INTRODUCTION

Skin grafting as a reconstructive procedure has many benefits, including accelerating the healing of burns and other wounds, and correcting scar contractures. Harvest of split-thickness skin graft causes damage in epidermal and dermal layers of skin at the donor site. The management of the donor site after harvesting a split-thickness skin graft is an important issue, as patients often report more discomfort at the donor site than at the recipient site.^1^ Various types of dressing methods have been used for skin donor sites to provide earlier regeneration.^2^^- ^^10^ The ideal split-thickness skin graft donor-site dressing would promote healing, cause minimal pain to the patient, prevent infection, result in minimal scarring, and be in- expensive and easy to use. A dressing which possesses all of these qualities has yet to be developed, but currently many dressing methods meet some of these criteria to varying degrees. These dressings can be grossly categorized into moist and non-moist dressings. The key difference is that moist dressings can prevent exudate desiccation by retaining moisture. In our plastic, reconstructive and burn unit, paraffin gauze dressing in a semi open manner is standard skin graft donor site dressing. When compared to dry fine mesh gauze dressing, paraffin gauze dressing was found to promote more rapid and more complete re- epithelialization, and to be a less painful donor site dressing.^11^ Also, patients have reported less pain during dressing removal with the paraffin gauze dressing than with dry fine mesh gauze dressing.^12^ Nitrofurazone soaked fine mesh gauze moist-healing, antimicrobial, and non-adherent properties suggest that it might be a good donor site dressing. However, there are no human studies in the literature which assess nitrofurazone soaked fine mesh gauze as a donor site dressing. Paraffin gauze and nitro- furazone gauze and dry gauze have for years been used by plastic surgeons in our ward for the coverage of split-skin donor sites.

The purpose of our study was to determine the best method of dressing the donor site split-thickness graft among three different methods with respect to the rate of healing, pain, secretion, infection and cost. We were particularly interested in this comparison of two moist healing regimens in addition of moist dressings against dry dressings. Since many donor site studies in the literature have compared healing of moist dressings against dry dressings,^3^^,^^12^^-^^14^ and most of these studies only confirm that re- epithelialization is retarded in a dry environ ment.^3^^,^^12^^,^^13^

## MATERIALS AND METHODS

The study was approved by the Institutional Review Board and informed consent was obtained from each patient who enrolled in the study. Sixty patients underwent split thickness skin grafting at our plastic, reconstructive and burn center were randomly divided into three groups based on sequential referral for operation (1-3) included in the study. All patients were operated upon under general anesthesia for coverage of skin defects. Exclusion criteria contained the patients who were not able to continue cooperation with the research group, the patients who were not able to estimate amount of the pain (Psychotics, Neurotics and Debilitates) and the patients who had medical therapy with drugs that may have change amount of pain sensation.

Donor site was the same for almost all of the patients (posterolateral or anterolateral thigh) and had not been used before. Thickness of the graft was the same for all patients. The grafts were harvested in a standard manner at 0.36–0.43 mm thickness and the donor sites were dressed with one of the following methods:

- Method A: Paraffin fine mesh gauze and intermediate layer of sterile plastic sheet witch was covered with 10 layers of dry gauze.

- Method B: Nitrofurazone soaked fine mesh gauze and intermediate layer of sterile plastic sheet witch was covered with 10 layers of dry gauze.

- Method C: Dry fine mesh gauze and inter- mediate layer of sterile plastic sheet witch was covered with 10 layers of dry gauze.

We used a side-by-side matched pair design by equally dividing the donor site to proximal and distal half for application of dressings to compare Method A and Method B in 20 pa- tients (Group 1), Method A and Method C in 20 patients (Group 2), Method B and Method C in 20 patients (Group 3). Thirty seven of the patients were men and 23 were women. The mean age of the patients in groups was 26.2 to 28.5 years (total mean of 27.2 years). After the operation, patients were hospitalized in our center, donor sites were not opened for two days. On the third day after operation, the outer layer dressings of the donor site and inter- mediate layer of sterile plastic sheet were easily taken off to examine the wound and to complete the chart. None were discharged before removal of intermediate layer of sterile plastic sheet and 10 layers of dry gauze over it. Only the first layer (paraffin gauze in A and nitrofurazone gauze in B and fine dry gauze in method C) remained attached to the wound. If there was obvious secretion on the remained first layer gauze, we used hair dryer to dry it and reduce secretions. Secretion of wound during the first 24 hours after removal of dressing was considered usual and was present in all of the cases. In patients that secretion of wound was obvious 24 hours after removal of superficial layers of dressing and we used hair dryer to dry it and reduce secretions it was considered positive for unusual secretion. Wounds were observed daily by the observer for signs of infection such as erythema, sever pain, induration, purulent discharge, and malodor. In suspicious cases a swab of wound was sent for routine culture and sensitivity.

**Fig. 1:**
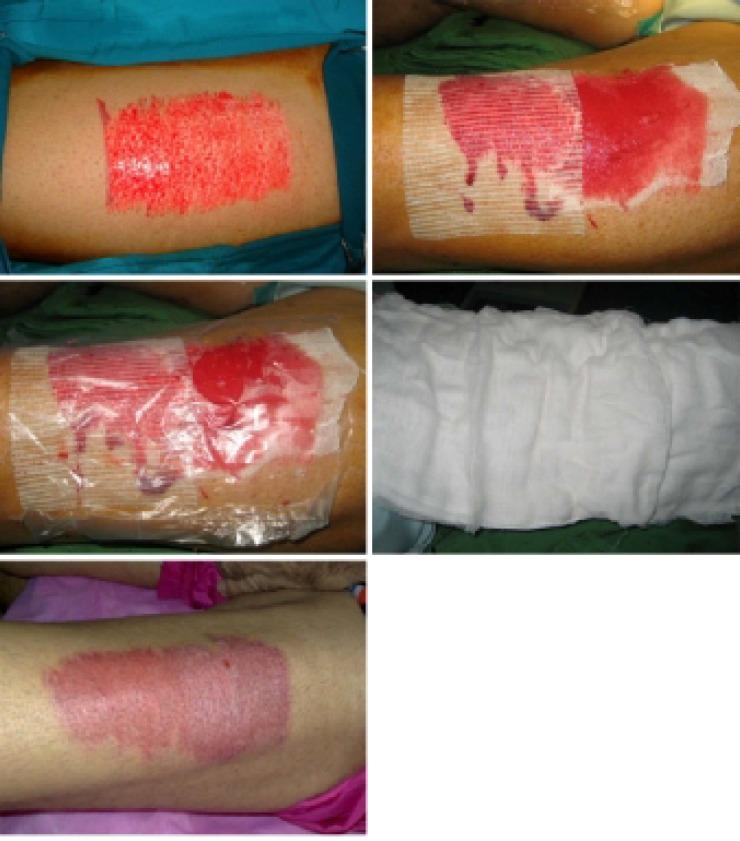
Dressing method of donor site. (Above left) Donor site wound. (Above right) First layer of dressing with method A&C. (Center left) Intermediate separating plastic sheet is applied. (Center right) Ten layers of fine mesh gauze is applied. (Below) Ten days after operation

In any method, waiting for spontaneous epithelialization of the wound, spontaneous detachment of gauze followed without any interference for detaching them. Repair time (the complete epithelialization of the wound) was documented by photography of the wound and recorded (Figure 1). A chart was used to note epithelialization, secretion status and infection. To evaluate pain objectively, visual analog pain scale was used.^15^ We asked patients to score the pain in donor site from 0 (no pain) to 10 (maximal pain), and it was recorded 1, 2, 3, 7, 14, 21 days after the operation. The cost of the materials used for dressing was the same in the three methods. Exceptions were the first layers of fine mesh gauze.

All results were analyzed using SPSS (Statistical Package for Social Sciences for Windows 13.0, Chicago, IL, USA) program. ANOVA test was used to analyze demographic data. Mann–Whitney U and Kruskal-Wallis tests were used to analyze differences between variables. Statistical analysis of the repair time performed by Kruskal-Wallis test to differentiate between methods. When analyzing variables descriptive statistics (mean and standard deviation) were used. Results were evaluated in 95% confidence intervals and significance was ascribed to a p-value <0.05.

## RESULTS

**Fig. 2:**
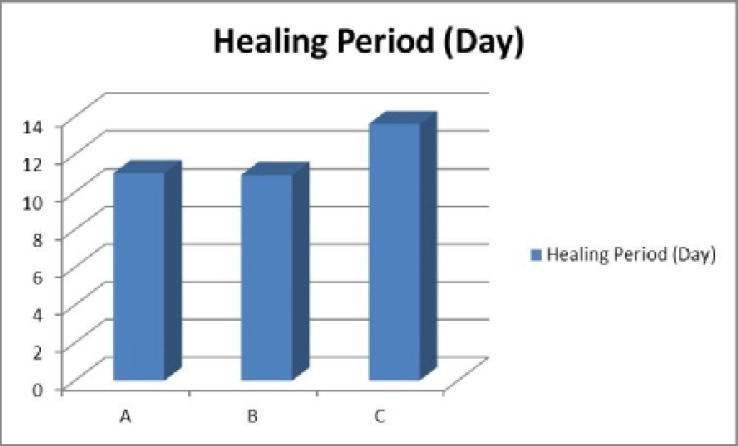
Duration of complete healing process in deferent methods

There was no statistical significant difference between average of age and sex in the groups (Table 1). Epithelialization time for method A was 11.2 days (SD=2.5). It was 10.9 days (SD=2.4) for method B (nitrofurazone soaked gauze) and 13.6 days (SD=3.4) for method C (dray gauze). There was a significant difference between three methods in average time of repair and dressing with nitrofurazone had the least time of repair (p<0.05) (Figure 2). Epithelialization was completed in all cases and complications such as infection or bleeding was not seen (Table 2).

**Table 1:** Demographic data of the patients

**Group**	**Mean age (Year)**	**No. of males (n=37)**	**No. of females (n=23)**
1	27.1	12	8
2	28.5	14	6
3	26.2	11	9
Total	27.2		
	P=0.3	P=0.55	

**Table 2:** Average time for epithelialization for methods

**Method**	**No.**	**Healing Period (Day)**
A	40	11±2.5
B	40	10.9±2.4
C	40	13.6±3.4
Total	120	11.9±3.07
		P=0.03

**Fig. 3:**
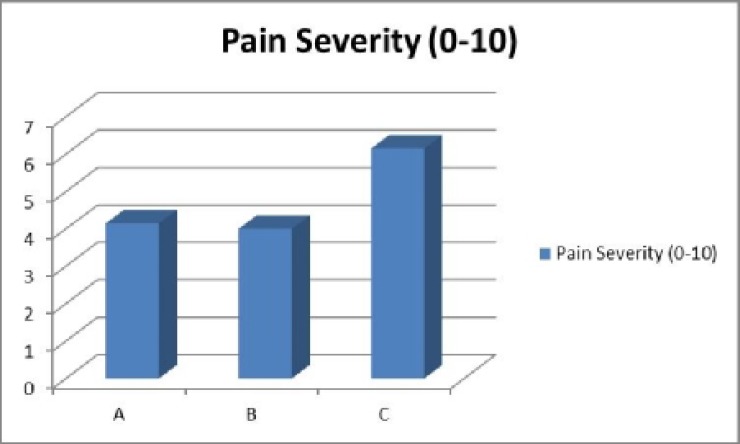
Comparison of Pain severity scores as recorded by patients

**Fig. 4:**
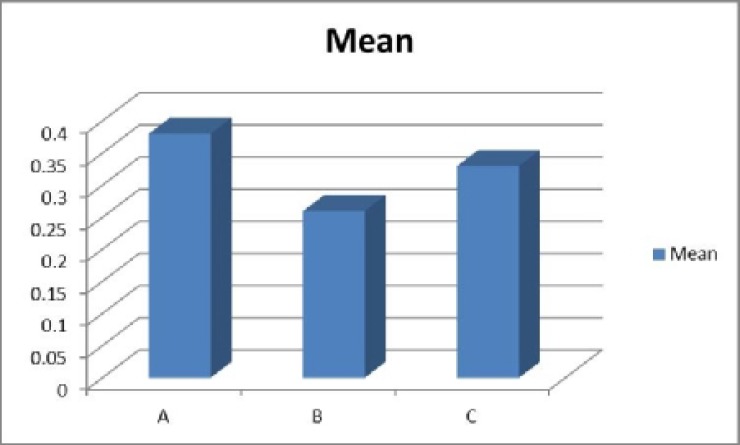
Comparison of average amount of secretion between methods

The mean values for the pain scores was 4.15 (SD=1.9) in Method A, 4 (SD=1.6) in Method B, and 6.15 (SD=1.5) in Method C (Table 3). Pain severity was the least in Method B (nitrofurazone soaked gauze) and statis tical difference between the groups was significant (p<0.05) (Figure 3). Average amount of unusual secretion was in 17% of patients (SD=38%) in Method A, 7% (SD=26%) in Method B, and 12% (SD=33%) in Method C (Table 4). There was a statistically significant difference between three methods and dressing with nitrofurazone had the least secretion (Figure 4). There was no statistically significant difference in cost of the management.

**Table 3:** Comparison of pain severity score for methods

**Method**	**No.**	**Pain severity (0-10)**
A	40	4.15±1.9
B	40	4±1.6
C	40	6.15±1.5
Total	120	4.7±1.9
		P<0.05

**Table 4:** Comparison of the rate of secretion between methods

**Method**	**No.**	**Secretion rate**
A	40	0.38±0.07
B	40	0.26±0.07
C	40	0.33±0.12 P<0.05

**Fig. 5:**
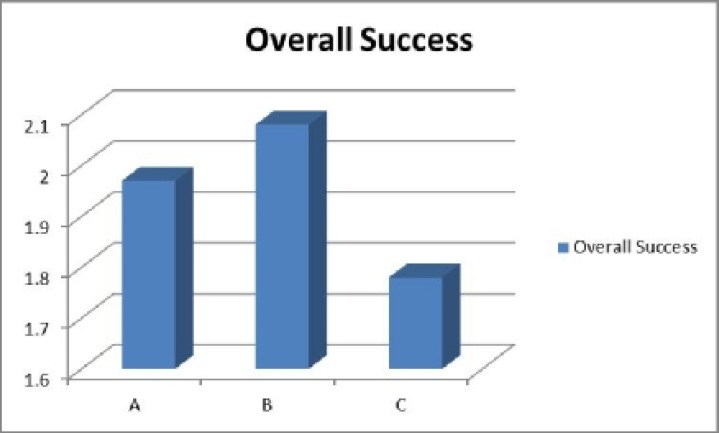
Overall success of management between methods

According to Figure 5, based on criteria that have been evaluated successful rate of dressing with Method B were more than two other methods. When challenged in two-by- two comparisons highly significant differences were shown in favor of Method B in comparison with Method C, but only borderline significance with Method A.

## DISCUSSION

Skin grafting has many benefits, including accelerating the healing of burns and other wounds, and correcting scar contractures.^1^^,^^16^ After skin graft harvest, the donor site becomes like a second degree burn injury which loses all epidermis and part of dermis. It is obvious that donor site of graft can cause pain, morbidity, hypertrophic scar, and even keloid. If donor site be- comes infected, it can lead to full thickness defect which is equal to third degree burn. The Purpose of the donor site management is to promote healing as quickly as possible without complications. Among the goals, relief of donor-site pain is probably the most important component from the patient’s point of view. Although the open approach, a donor site without dressing costs much less than closed approaches, it is painful and takes longer time for healing.^5^ Epithelial cell proliferation and migration are believed to be optimal in a moist environment.^17^^- ^^19^

Many dressing methods and medications have been reported for split thickness donor site management but a consensus has not yet been formed.^2^^-^^7^^,^^20^ Barnea *et al. *compared paraffin gauze with Aquacel and reported that results with Aquacel were superior to paraffin gauze. However, cost-effectiveness of this ma- terial was discussed in this study.^6^ Disa *et al. *used combined calcium sodium alginate and bio occlusive membrane dressing alternative to paraffin gauze in the management of split- thickness skin graft donor sites. They reported good results but it was more expensive.^7^

Paraffin gauze and nitrofurazone gauze and dry gauze have for years been used by plastic surgeons in our ward for the coverage of split- skin donor sites, the aim of our study was determining a method which cause rapid repair and has least complications such as bleeding, pain, infection and secretion, and is more economic. In this study, 60 patients who needed split thickness graft for treatment of wounds, were divided into three groups randomly. Each group had 20 persons and we used two different methods of dressing in each person, so we had 40 samples for each method. There was no statistically significant difference in demographic data, such as sex, age and educational level. Each method of dressing was examined for pain, epithelialization time, infection and secretions. Statistical analysis of data revealed that dressing with nitrofurazone soaked gauze had the least repair time, pain and secretion. This results in conclusion that to oil the donor site reduces epithelialization time. As oiling prevents draying of dermal tissue and deep tissues, it keeps them moist, and we know that repair of partial thickness wounds is faster in the moist environment. Moist environment causes quick migration of keratinocytes to the wound and epithelialization. Antibiotic ointment moistening not only reduces the risk of infection and prevents disturbance in repair process, but also, as it’s oily, moistens the environment and accelerates repair process by reducing bacterial activity, so reduces the depth of scar and this may be the reason of reduction in pain by nitrofurazone use. Although additional drug application seems like increase the cost of the management, it reduces the cost by decreasing hospitalization time. We believe that widespread studies is necessary to confirm our findings, this study can lead to more widespread studies.

## CONFLICT OF INTEREST

The authors declare no conflict of interest.

## Footenote

Please cite this paper as:

Hassanpour SE, Moosavizadeh SM, Yavari M, Hallaj Mofrad HR, Fadaei AR. Comparison of Three Different Methods of Dressing for Partial Thickness Skin Graft Donor Site. World J Plast Surg 2013;2(1): 26-32.
